# Fatal Spinal Cord Edema Following Cervical Decompression Surgery Possibly Associated with Dialysis Disequilibrium Syndrome: A Case Report

**DOI:** 10.31662/jmaj.2025-0360

**Published:** 2025-12-05

**Authors:** Kazuhiro Horiuchi, Shuntaro Nakamura, Kazuki Yamada

**Affiliations:** 1 Department of Neurology, Hakodate Municipal Hospital, Hakodate, Japan

**Keywords:** dialysis disequilibrium syndrome, spinal cord edema, hemodialysis, cervical spondylotic myelopathy, postoperative complication

## Abstract

Dialysis disequilibrium syndrome (DDS) is a rare neurological complication of hemodialysis caused by rapid osmotic shifts. While its cerebral effects are well recognized, its impact on the spinal cord has not been well documented. We report the fatal case of a 57-year-old man on maintenance hemodialysis who developed progressive spinal cord edema 15 days after posterior cervical decompression surgery. The patient’s neurological deterioration occurred in temporal association with each hemodialysis session, during which the patient exhibited significant fluctuations in blood urea nitrogen (pre-dialysis range: 54.8-122.9 mg/dL). Despite aggressive management, including extended dialysis and continuous renal replacement therapy, his condition worsened. Autopsy revealed extensive spinal cord necrosis at the surgical site, with findings consistent with severe, recurrent edema and its compressive effects, without evidence of a primary ischemic or inflammatory pathology. This case suggests a devastating, atypical manifestation of a DDS-like pathophysiology, where the surgically compromised spinal cord may have become a vulnerable target for osmotic shifts, underscoring the need for a high index of suspicion in post-neurosurgical patients undergoing hemodialysis.

## Introduction

Dialysis disequilibrium syndrome (DDS) is a serious neurological complication of hemodialysis caused by cerebral edema resulting from rapid post-dialysis serum urea reduction ^(1), (2)^. DDS occurs when urea is removed from the blood more quickly than it is cleared from the central nervous system ^(1), (3)^. Although DDS typically presents with cerebral symptoms, focal spinal cord edema has not, to our knowledge, been reported. We hypothesize that this represents a localized DDS variant in which a surgically compromised blood-spinal cord barrier (BSCB) served as the site of injury.

We report this fatal case to highlight a critical, previously unrecognized risk for patients on hemodialysis who undergo spinal surgery.

## Case Report

**Figure 1.**
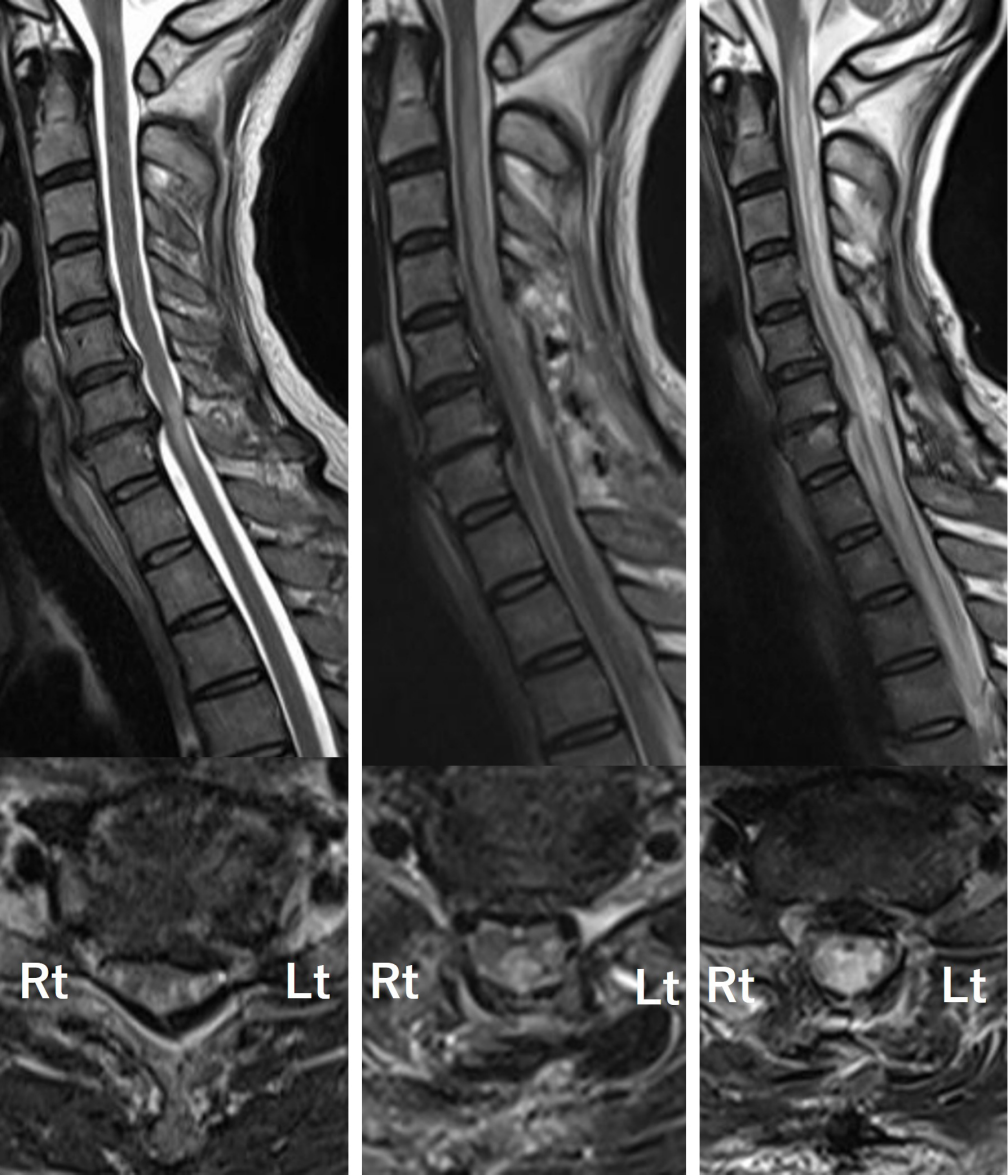
Upper row: Sagittal T2-weighted images of cervical spine MRI over time. Lower row: Axial T2-weighted images at the C6/7 level. A. Preoperative cervical MRI. Signal changes due to spinal cord compression are observed at the C6 and C7 levels, findings consistent with cervical spondylotic myelopathy. B. Cervical MRI 15 days postoperatively. The images show sustained decompression of the spinal cord; however, new T2-weighted hyperintensity lesions are observed in the dorsal columns, extending from the lower medulla to the C7 level. C. Cervical MRI 45 days postoperatively. Despite treatment with intravenous methylprednisolone, the lesions were resistant to therapy. The patient’s symptoms progressively worsened after each dialysis session, which correlated with the expansion of abnormal signal intensity on MRI. MRI: magnetic resonance imaging.

A 57-year-old man with a five-year history of maintenance hemodialysis for nephrosclerosis presented with progressive myelopathy. The patient had been receiving hemodialysis three times a week for 3.5 hours per session, with an ultrafiltration volume of 3.5 L, using a polyethersulfone dialyzer (MFX-21SW eco; Nipro, Osaka, Japan). The patient underwent C4-T1 posterior decompression for cervical spondylotic myelopathy (Figure 1A). The surgery was uneventful, and the patient initially improved.

On postoperative day 15, the patient developed recurrent sensory deficits and gait disturbance. Neurological examination revealed decreased superficial sensation below the T9 dermatome and impaired proprioception. Cervical magnetic resonance imaging (MRI) showed a new T2-weighted hyperintensity in the dorsal columns, extending from the lower medulla to C7 (Figure 1B). An extensive workup, including serology for autoimmune myelopathies and cerebrospinal fluid analysis, was negative for infectious or inflammatory causes.

**Figure 2.**
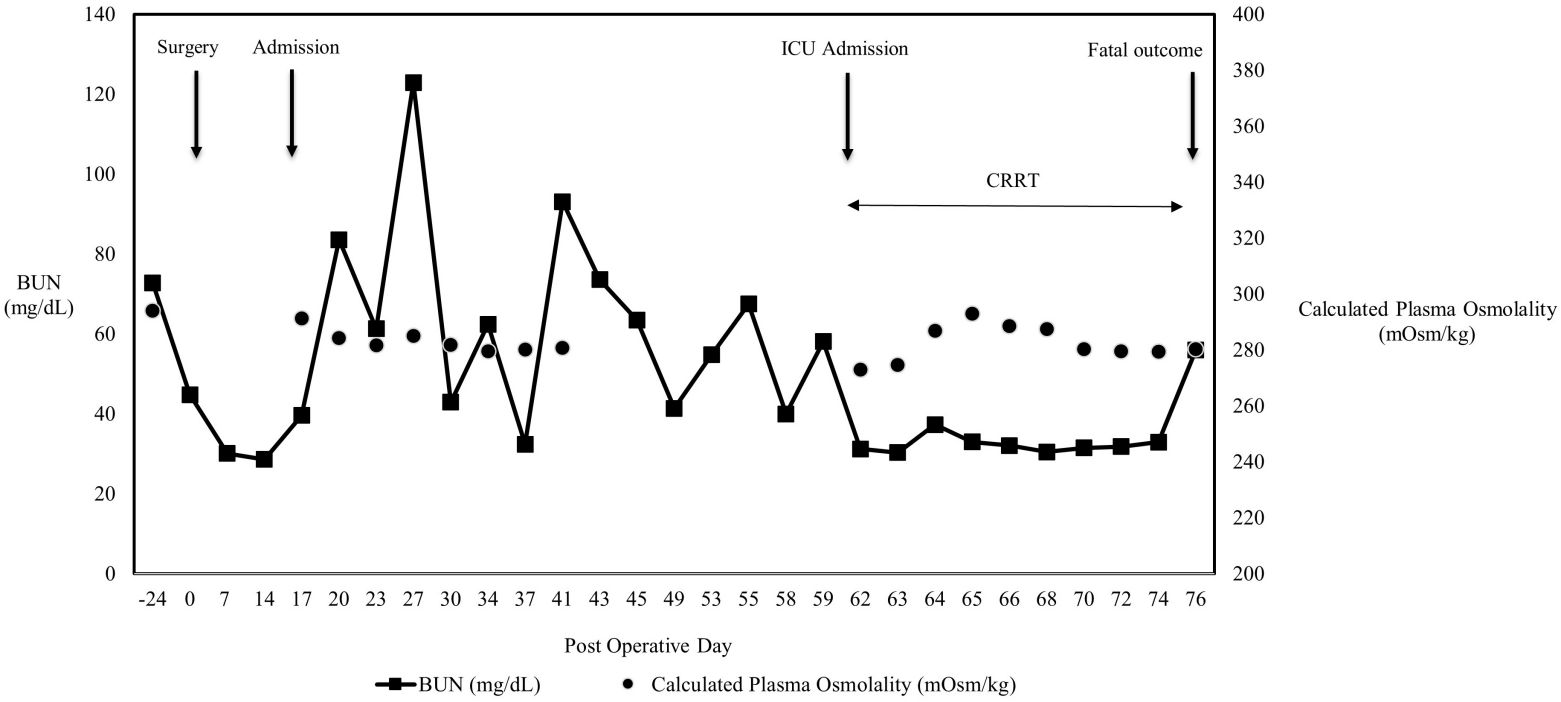
Trends in BUN and Calculated Plasma Osmolality Correlated with Clinical Events. The graph shows the trend of BUN (solid line) and calculated plasma osmolality (circles) throughout the patient’s postoperative course. The cyclical peaks in BUN prior to intermittent hemodialysis demonstrate a large, recurring osmotic gradient. Note that the less pronounced decrease in calculated plasma osmolality may be partially masked by corticosteroid-induced hyperglycemia. Key clinical events, including the day of surgery (Day 0), hospital admission for neurological decline (Day 15), initiation of CRRT upon ICU admission (Day 62), and fatal outcome (Day 74), are indicated by arrows. BUN: blood urea nitrogen; CRRT: continuous renal replacement therapy; ICU: intensive care unit.

Despite treatment for suspected immune-mediated myelopathy with plasma exchange and intravenous methylprednisolone, his condition worsened after each hemodialysis session. Follow-up MRI showed progression of the lesion (Figure 1C). A retrospective review of his laboratory data revealed stable serum sodium but with wide fluctuations in blood urea nitrogen (BUN) levels. Pre-dialysis BUN ranged from 54.8 to 122.9 mg/dL (Figure 2). His fatigue, headache, and sensory disturbances consistently worsened during and after hemodialysis.

**Figure 3.**
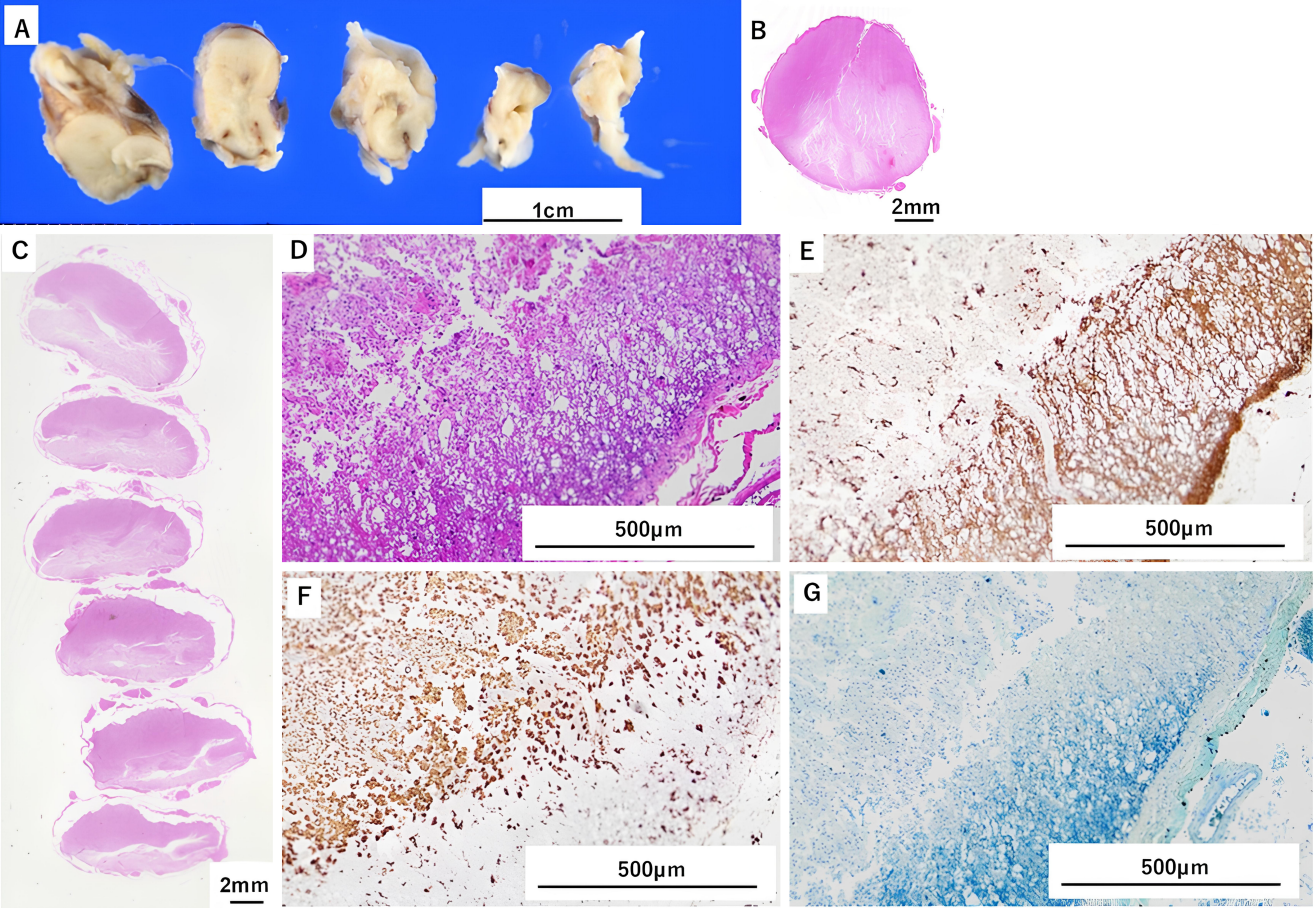
A. Gross pathological findings of the medulla oblongata and cervical spinal cord. Necrosis-induced liquefaction was observed on the dorsal aspect of the medulla and cervical spinal cord. The affected areas showed severe necrosis and atrophy, appearing grayish-white to pale yellowish-white, with noticeable softening and volume loss. B (medulla), C (cervical spinal cord). Axial sections of the medulla oblongata and cervical spinal cord (HE staining). Observations revealed a marked reduction in neuronal density, predominantly on the dorsal side. D (medulla), E (cervical spinal cord). Low-magnification microscopic views (HE staining). Microscopic examination highlighted the loss of the myelin sheath, clustering of macrophages, astrocytic proliferation in response to damage, and degeneration and loss of nerve fibers. Numerous gaps were noted, likely due to the disappearance of necrotic cells.

To mitigate osmotic shifts, dialysis duration was extended, but his neurological status continued to deteriorate. On postoperative day 60, the patient developed altered consciousness and respiratory failure, requiring continuous hemodiafiltration. His course was further complicated by sepsis and multiorgan failure, leading to death on postoperative day 74. Autopsy revealed extensive necrosis and atrophy in the dorsal column without significant inflammation, hematoma, or vascular malformation (Figure 3).

## Discussion

We describe a fatal case of progressive spinal cord necrosis following cervical surgery in a chronic hemodialysis patient. We propose a localized variant of DDS, recognizing that causality remains inferred. The inference is based on a convergence of evidence: (i) Reproducible neurological deterioration time-locked to each hemodialysis session, (ii) The exclusion of other major differential diagnoses by extensive workup and autopsy, and (iii) The focal nature of the edema at the surgically compromised site.

Unlike cerebral DDS, which causes global encephalopathy (headache, seizures, altered consciousness) ^(1), (3)^, our patient developed focal myelopathy. The spinal cord’s confinement within the narrow canal may render it particularly vulnerable to even mild edema, predisposing it to necrosis.

Ischemia-reperfusion injury can also cause spinal edema but typically induces an early inflammatory response in animal models ^(4)^. In contrast, autopsy findings here showed necrosis with minimal inflammation. White cord syndrome, another reperfusion injury variant ^(5)^, generally presents immediately postoperatively ^(6)^, unlike this delayed course. Preoperative MRI revealed extensive T2 hyperintensity and mild swelling, findings atypical for simple compressive myelopathy. We postulate that chronic uremia induced subclinical cord vulnerability, explaining both preoperative imaging and the catastrophic postoperative response.

We further suggest that a compromised BSCB at the decompression site acted as the focal point of injury. BSCB disruption is increasingly recognized in degenerative cervical myelopathy and correlates with disease severity ^(7)^. Although decompression may promote recovery, transient postoperative hyperpermeability occurs ^(8)^. In this patient, chronic and surgical BSCB injury may have created a locus susceptible to DDS-like osmotic stress.

The absence of serial plasma osmolality is a limitation. Nonetheless, large BUN declines provide indirect evidence of a clinically significant gradient. Once severe spinal necrosis occurred, the damage proved irreversible despite prolonged dialysis, underscoring the importance of prevention.

In conclusion, DDS-like mechanisms may cause severe postoperative spinal cord injury. Clinicians should consider this pathophysiology in dialysis patients who develop new neurological deficits after spinal surgery. Proactive management, such as employing modified, gentler dialysis protocols (e.g., shorter duration, lower blood flow, or the use of mannitol) in the immediate postoperative period, may be critical to prevent this devastating complication.

## Article Information

### Acknowledgments

We thank the staff of the medical unit for their assistance in managing this patient.

### Author Contributions

Care of patients: Kazuhiro Horiuchi, Shuntaro Nakamura, and Kazuki Yamada. Manuscript writing, design, and editing: Kazuhiro Horiuchi. Critical review of the manuscript and approval of the final version: All authors.

### Conflicts of Interest

None

### IRB Approval Code and Name of the Institution

The Hakodate Municipal Hospital Institutional Review Board approved this case report (approval number: [2025-102]). Informed consent was obtained from the patient’s wife.
